# Chicago Dental Society

**Published:** 1885-02

**Authors:** A. E. Baldwin


### CHICAGO DENTAL SOCIETY.

### JANUARY MEETING.

REPORTED FOR THE INDEPENDENT PRACTITIONER BY A. E. BALDWIN, M. D.

The paper of the evening was by Dr. G. W. Nichols, and his subject was "Eclecticism as Applied to the Practice of Dentistry."

He said that following the able and truly "eclectic" paper presented at the last meeting by Dr. Talbot, it would seem almost superfluous for him to say much upon the subject. He defined eclecticism strictly as a method which, without having any fixed principles within itself, forms such by gathering, selecting, rejecting or combining, out of all other methods, that which will best subserve the demands of the case under consideration. The physician or dentist should meet his patient unbiased by "ism" or "pathy," and should consequently diagnose according to the history and condition of the case, and treat entirely in accordance with such condition. In brief, then, the subject of this paper means practicing dentistry solely in accordance with the indications in each and every case. From this view of the subject it has more to do with the mental condition of the dentist than with the action of medicaments, for a mistaken, narrow-minded, non-ethical treatment is of little avail. Each should be careful to know that his "light" is not "darkness." Man's best inheritance is spiritual and mental freedom. The training from childhood should be how best to utilize one's own powers; to acquire the habit of close, analytical observation; to educate more by drawing out than by absorbing the ideas of others. The majority get facts entirely by adoption; too often they are satisfied if they know what so-and-so thinks or does under like circumstances. Such men are not, neither can they be, eclectic.

The next great hindrance to attaining this perfection is egotism. Every man proceeds upon the idea that his methods and practices, or those taught to him, must, as a matter of course, be correct, and that all others are unworthy even a fair investigation. Especially is this true of egotistical teachers, who inculcate theories more or less false and unimportant, as ideas of great moment. He instanced the following: The practice of placing creosote on cotton in root canals, supposing that the contained septic matter would thus be rendered innocuous; capping putrescent pulps; drastic cathartics in inflammations of mucous tracts; replantation or transplantation of diseased teeth; the extensive cutting away of tooth structure for easy access to cavities, without subsequent restoration of contour; the use of cohesive foil in approximal surfaces of chalky teeth, or as an exclusive filling material; the total condemnation of grooves or retaining pits; filling sensitive teeth with metals without first relieving that condition; the use of hopelessly diseased roots for the support of artificial substitutes; the extraction of sound teeth on any pretext; non-removal of deposit upon roots; above all, the sinful and culpable silence of practitioners regarding hygienic observations on the part of their patients, and the pretense that their methods are the only invariably successful ones.

The dentist who is not master of all methods really has none. Many who thrive financially will never accomplish much good. Boasted methods fail in very many cases, and it is only by the exercise of an enlightened and clear judgment that the best shall be perceived and applied. Each individual case brings its own needs, and out of it should be gathered the plans for successful treatment.

This is eclecticism in dentistry and in medicine.

DISCUSSION.

*Dr. Noyes*—While commending the paper, believes the principal difficulty will prove to be in the attempt to learn all methods, to enable one to choose the best, as in the use of medicaments, materials, implements, etc.

*Dr. Allport*—Commends the paper—compares our professional beliefs to political faith—in that one may honestly believe what to another is totally wrong. To one the use of soft gold is a great error; to another cohesive gold is a mistake. Thinks we are all too liable to be influenced by prejudice, or preconceived notions.

*Dr. Marshall*—Believes that the successful practice of dentistry lies in true eclecticism. As you all know, fifteen years ago those who used amalgam were tabooed. Now, on every hand, we see plastics, tin, and amalgams used, and, when rightly used, much good resulting therefrom. Many failures are not because of poor operations, but because of improper selection of material. We should not be governed by inflexible rules. We must not use all gold, or all amalgam; must not separate solely with the file, nor restore all by contour.

*Dr. Koch*—Disagreed with Dr. Marshall in regard to the total discountenance of amalgam, especially in Chicago, and firmly believes that dentists should have a reason for all things, and thus be a true eclectic.

